# Spirometric Assessment of Pulmonary Function Tests in Asthma Patients

**DOI:** 10.7759/cureus.54979

**Published:** 2024-02-26

**Authors:** Mitali B Rathod, Amal Budensab, Sarvasv Bhalla, Neethi Kavi Mahesh, Elizabeth Alex, Mariam Jesudas

**Affiliations:** 1 Department of General Medicine, NAMO Medical College and Research Centre, Silvassa, IND; 2 Department of General Medicine, SDM (Shri Dharmasthala Manjunatheshwara) College of Medical Sciences and Hospital, Dharwad, IND; 3 Department of General Medicine, Krishna Vishwa Vidyapeeth, Malkapur, IND; 4 Department of General Medicine, M. S. Ramaiah Medical College, Bengaluru, IND; 5 Department of General Medicine, J.J.M. Medical College, Davanagere, IND; 6 Department of General Medicine, Pushpagiri Medical College Hospital, Tiruvalla, IND

**Keywords:** obesity, females, pulmonary function test, spirometry, asthmatics

## Abstract

Background and aims: Asthma is a chronic airway inflammatory disorder that imposes substantial morbidity and mortality. Spirometry is a significant tool for the objective measurement of obstruction among asthmatics. The present study was conducted to assess the pulmonary function test parameters among asthmatics and compare the observed and predicted values.

Materials and methods: This cross-sectional research was performed on 120 asthmatic patients who attended a tertiary care healthcare center and underwent spirometry evaluation. The spirometry indices such as forced vital capacity (FVC), forced expiratory volume in one second (FEV1), FEV1/FVC ratio, peak expiratory flow rate (PEFR), and maximal voluntary ventilation (MVV) were recorded. Further, a gender-wise comparison of spirometry indices was also done.

Results: There was a substantial decrease in FVC (2.05 ± 0.12 vs. 2.75 ± 0.24 L/sec; p = 0.02), FEV1 (1.78 ± 0.16 vs. 2.38 ± 0.32 L/sec; p = 0.01), FEV1/FVC ratio (74 ± 4.38 vs. 83 ± 5.76 %; p = 0.01), PEFR (4.76 ± 0.42 vs. 5.82 ± 0.65 L/sec; p = 0.03), and MVV (78.65 ± 28.45 vs. 115.87 ± 32.15 L/min; p = 0.001) for observed and predicted values. Female asthmatic patients displayed a substantial decline in FVC (p = 0.001), FEV1 (p = 0.006), FEV1/FVC (p = 0.001), and MVV (p = 0.01) when compared to males.

Conclusion: This study suggests that asthmatic individuals had impaired lung function upon initial assessment. Female asthmatic patients studied are at increased risk of asthma severity when compared to males.

## Introduction

Asthma is the most common non-communicable disease characterized by persistent inflammation of the airways, resulting in the constriction of the small air passages in the lungs. The classical indications of asthma comprise wheezing, chest tightness, cough, and shortness of breath, and may vary in severity and duration. Asthma has several clinical forms, such as allergic, non-allergic, late-onset, and asthma with persistent airflow limitation [1]. Globally, around 262 million people were affected with asthma in 2019, with a mortality estimate of 461.07 thousand annually [2]. In 2019, the global prevalence of asthma was estimated to be 11.5% in people aged five to 69 years [3]. According to the Global Burden of Disease 2019 estimates, the prevalence of asthma in India is estimated to be 34.3 million, which accounts for 13.09% of the global burden [4].

The diagnosis of asthma can be established by assessing the patient's symptoms and physical examination findings, as well as by evaluating the reversible airflow obstruction and airway hyperresponsiveness. Spirometry is the standard technique to assess lung function and determine the severity of asthma in individuals of all ages [5]. Albeit, the results of the pulmonary function test (PFT) are not of diagnostic importance, but any deviation in the results will help in confirmative diagnosis in a wide range of respiratory pathologies, including asthma, and it can be correlated with the patient's history [6].

Mounting studies indicate that in asthmatic patients, airflow obstruction is not completely reversible and the majority of the individuals undergo a rapid and continuous decline in lung function [6,7]. Patients with asthma experience significantly more pronounced decreases in forced expiratory volume in one second (FEV1), FEV1/forced vital capacity (FVC) ratio, and peak expiratory flow rate (PEFR) over a period of time as compared to non-asthmatic individuals [8]. Further, in the majority of asthmatic patients, the values of FEV1 and PEFR are less than 80% of the predicted value. Furthermore, it was found that the results were below 80% of the predicted levels [9]. Maximal voluntary ventilation (MVV) is also reduced in the majority of asthmatic individuals and thus serves as a reliable indicator for predicting the presence of asthma [10]. In addition, proportional reductions in the FEV1 and FVC are associated with increased cardiovascular comorbidity and mortality [11,12]. The clinical expression of asthma is dependent on various mechanisms, such as sex hormones, obesity, exposure type, and age at onset [13]. Females had a higher prevalence of uncontrolled asthma and respiratory symptoms and worsened self-reported quality of life as compared to males [14]. Against this backdrop, the aim of this study was to assess the PFT indices among asthmatics and compare the observed and predicted values. Further, the differences between the males and females in terms of PFT indices were also evaluated.

## Materials and methods

This was a cross-sectional study conducted on 120 asthmatic patients attending the Department of General Medicine from January 2022 to December 2022. The study was approved by the Institutional Ethical Committee of Namo Medical Education & Research Institute, Silvassa (IEC/2021/312/176) and informed consent was obtained from all the patients.

Inclusion criteria

Individuals aged more than 18 years with a diagnosis of asthma made by physicians were included in the study. The diagnosis of asthma was made by the following criteria: asthma is characterized by reversible obstruction of airways, which is accompanied by a 12% increase and 200 ml in FEV1 post-short-acting β2 agonist administration or the presence of bronchial hyperresponsiveness after giving bronchoconstriction drug such as methacholine [15].

Exclusion criteria

Patients with chronic lung pathologies other than asthma, such as chronic obstructive pulmonary disease and bronchiectasis, were excluded from the study. Patients who underwent lobectomy, had abnormal chest X-rays, and were pregnant were also excluded from the study. Patients suffering from chronic illnesses that have the potential to impact their functional status, such as ischemic heart disease or cardiac failure, were also excluded from the study

The severity of asthma was assessed following the Global Initiative for Asthma (GINA) guidelines and categorized as mild, moderate, and severe based on daily medical use [15]. The PFT was done using a digital computed spirometer (Sensormedics, Anaheim, CA) according to the recommendations of the American Thoracic Society (ATS). The test was conducted after withholding a short-acting inhaled beta-agonist for at least a minimum of six hours. The patients were allowed to sit comfortably and explained about the spirometry machine and then the procedure was carried out. The best of three values of FVC, FEV1, FEV1/FVC ratio, and PEFR were recorded. The MVV test involves instructing the individuals to inhale deeply (maximum inspiration) and then forcefully exhale and completely empty their lungs (maximum expiration) as quickly and forcefully as they can for a duration of 10 seconds. The spirometry was conducted by trained staff, and the equipment adhered to the ATS standard for precision and accuracy.

Data analysis

IBM SPSS Statistics for Windows, version 25.0 (IBM Corp., Armonk, NY) was used for the statistical analysis of the data. The continuous variables were mentioned as mean ± SD and the categorical variables were denoted as frequency (%). The difference in predicted and observed values of spirometry variables was associated with paired Student's t-test. The gender-wise comparison of variables was done using unpaired Student's t-test and chi-square analysis. P-value <0.05 was considered as statistically significant.

## Results

This study was conducted between January 2022 and December 2022 and we have evaluated 120 patients with asthma. The demographics and clinical characteristics of the subjects are depicted in Table 1. The mean age of the subjects was 54.17 ± 12.34 years. Female preponderance was observed with 60% being females. Regarding smoking status, the majority of the patients were never smokers (56.7%) and 43.3% were former smokers. The mean BMI among the asthmatic patients was 29.12 ± 4.76 kg/m2 and the majority of the patients (48.3%) were obese. Based on the asthma severity, 82 (68.4%) had severe asthma, 22 (18.3%) had moderate asthma, and 16 (13.3%) had mild asthma.

**Table 1:** Demographics and clinical characteristics of asthma patients GINA: Global Initiative for Asthma; BMI: body mass index.

Variables	Study participants (n = 120)
Age in years (mean ± SD)	54.17 ± 12.34
Age at diagnosis in years (mean ± SD)	25.76 ± 3.65
Gender (n, %)	
Male	48 (40%)
Female	72 (60%)
Smoking status (n, %)	
Never smoker	68 (56.7%)
Former smoker	52 (43.3%)
BMI, kg/m^2^ (mean ± SD)	29.12 ± 4.76
BMI category (n, %)	
Normal weight	24 (20%)
Overweight	38 (31.7%)
Obese	58 (48.3%)
GINA asthma severity classification (n, %)	
Mild	16 (13.3%)
Moderate	22 (18.3%)
Severe	82 (68.4%)

The spirometry pulmonary measurements of normal predicted value and observed value among the asthma patients are shown in Table 2. There was a substantial decline in the pulmonary function in observed value when compared to the normal predictive values, such as FVC (2.05 ± 0.12 vs. 2.75 ± 0.24 L/sec; p = 0.02), FEV1 (1.78 ± 0.16 vs. 2.38 ± 0.32 L/sec; p = 0.01), FEV1/FVC (74 ± 4.38 vs. 83 ± 5.76 %; p = 0.01), PEFR (4.76 ± 0.42 vs. 5.82 ± 0.65 L/sec; p = 0.03), and MVV (78.65 ± 28.45 vs. 115.87 ± 32.15 L/min; p = 0.001), respectively.

**Table 2:** Spirometry measurement of predicted and observed values among asthma patients The data are shown as mean ± SD. The association between predicted and observed values was done using the paired Student's t-test. * denotes p-value < 0.05 and it was statistically significant. FVC: forced vital capacity; FEV1: forced expiratory volume in one second; PEFR: peak expiratory flow rate; MVV: maximal voluntary ventilation.

Parameters	Predicted value (mean ± SD)	Observed value (mean ± SD)	P-value
FVC (L/sec)	2.75 ± 0.24	2.05 ± 0.12	0.02*
FEV1 (L/sec)	2.38 ± 0.32	1.78 ± 0.16	0.01*
FEV1/FVC (%)	83.62 ± 15.76	74.46 ± 17.38	0.01*
PEFR (L/sec)	5.82 ± 0.65	4.76 ± 0.42	0.03*
MVV (L/min)	115.87 ± 32.15	78.65 ± 28.45	0.001*

The gender-wise comparison of demographics and clinical characteristics are shown in Table 3. In this study, the age at diagnosis was lower in females as associated with males and it was significant (35.76 ± 3.76 vs. 39.65 ± 4.12; p = 0.02). Meanwhile, the BMI (31.76 ± 3.65 vs. 27.98 ± 2.42; p = 0.001) and the incidence of obesity were higher in females as compared to males (51 (70.8%) vs. 7 (14.6%); p = 0.002). Further, there was no significant difference in the asthma severity between females and males (p = 0.08), but the frequency of severe asthma was higher in females as associated with males.

**Table 3:** Gender-wise comparison of demographics and clinical characteristics of asthma patients The comparison was made between females and males. ^a^ Unpaired Student's t-test; ^b^ chi-square test; * denotes p-value < 0.05 and it was statistically significant. NS: non-significant; GINA: Global Initiative for Asthma; BMI: body mass index.

Variables	Females (n = 72)	Males (n = 48)	P-value
Age in years (mean ± SD)	55.76 ± 11.87	54.28 ± 12.76	0.65^a,NS^
Age at diagnosis in years (mean ± SD)	35.76 ± 3.76	39.65 ± 4.12	0.02^a,^*
Smoking status (n, %)			
Never smoker	55 (76.4%)	13 (27.1%)	0.001^b,^*
Former smoker	17 (23.6%)	35 (72.9%)	
BMI, kg/m^2^ (mean ± SD)	31.76 ± 3.65	27.98 ± 2.42	0.001^b,^*
BMI category (n, %)			
Normal weight	6 (8.4%)	18 (37.5%)	0.002^b,^*
Overweight	15 (20.8%)	23 (47.9%)	
Obese	51 (70.8%)	7 (14.6%)	
GINA asthma severity classification (n, %)			
Mild	7 (9.7%)	9 (18.7%)	0.08^b,NS^
Moderate	10 (13.9%)	12 (25%)	
Severe	55 (76.4%)	27 (56.3%)	

A gender-wise comparison of spirometry pulmonary functions is shown in Table 4. In this study, within the genders, there was a substantial decline in FVC (p = 0.001), FEV1 (p = 0.001), FEV1/FVC (p = 0.001), and MVV (p = 0.001) when compared between predicted and observed values among females. Likewise, in males, there was a significant decrease when comparing predicted and observed values for FEV1 (p = 0.01), PEFR (p = 0.01), and MVV (p = 0.001), while for FVC (p = 0.65) and FEV1/FVC (p = 0.76), it was not significant. When compared between males and females, there was no substantial difference in the predicted value for FVC, FEVI, FEV1/FVC, and PEFR, except for MVV (p = 0.001). Meanwhile, the observed value was significantly lesser in females as associated to males for FVC (2.15 ± 0.32 vs. 3.18 ± 0.93 L/sec; p = 0.001), FEV1 (1.62 ± 0.18 vs. 2.04 ± 0.97 L/sec; p = 0.006), FEV1/FVC (76.54 ± 17.12 vs. 81.32 ± 19.28%; p = 0.001), and MVV (71.65 ± 15.28 vs. 89.12 ± 17.43 L/min; p = 0.01). Meanwhile, the observed value of PEFR was lower in males as compared to females (2.54 ± 0.16 vs. 4.78 ± 0.45; p = 0.001) and it was significant.

**Table 4:** Gender-wise comparison of spirometry pulmonary functions among asthma patients The data are shown as mean ± SD. ^a ^The association between predicted and observed values between genders was performed by unpaired Student's t-test. ^b^ Comparison between predicted and observed values within the gender was done using paired Student's t-test. * denotes p-value < 0.05 and it was statistically significant. FVC: forced vital capacity; FEV1: forced expiratory volume in one second; PEFR: peak expiratory flow rate; MVV: maximal voluntary ventilation.

Parameters	Gender	Predicted value (mean ± SD)	Observed value (mean ± SD)	P-value
FVC (L/sec)	Female	2.72 ± 0.17	2.15 ± 0.32	0.001^b,*^
	Male	3.32 ± 0.46	3.18 ± 0.93	0.65^b,NS^
	P-value	0.07^a,NS^	0.001^a,*^	
FEV1 (L/sec)	Female	2.26 ± 0.28	1.62 ± 0.18	0.001^b,*^
	Male	2.58 ± 0.34	2.04 ± 0.97	0.01^b,*^
	P-value	0.13^a,NS^	0.006^a,*^	
FEV1/FVC (%)	Female	84.18 ± 18.36	76.54 ± 17.12	0.001^b,*^
	Male	83 ± 16.82	81.32 ± 19.28	0.76^b,NS^
	P-value	0.52^a,NS^	0.001^a,*^	
PEFR (L/sec)	Female	4.84 ± 0.67	4.78 ± 0.45	0.35^b,NS^
	Male	4.12 ± 0.82	2.54 ± 0.16	0.01^b,*^
		0.87^a,NS^	0.001^a,*^	
MVV (L/min)	Female	92.43 ± 18.76	71.65 ± 15.28	0.007^b,*^
	Male	132.65 ± 28.12	89.12 ± 17.43	0.001^b,*^
		0.001^a,*^	0.01^a,*^	

## Discussion

Bronchial asthma is a chronic inflammatory condition affecting the airways characterized by frequent episodes of wheezing, difficulty in breathing, tightness in the chest, and cough, which is further defined by bronchial hyperresponsiveness and variations in airflow obstruction, which subsides either spontaneously or with treatment. The treatment outcome depends on the reduction in symptoms without acute exacerbations; however, in the general population, there is a lack of effective asthma control. Various factors affect asthma treatment, such as smoking, female gender, and BMI [16]. PFT precisely evaluates the functional ability of the respiratory system, allowing us to identify the disease at an early stage and understand its progression and reaction to treatment. Various diseases exhibit distinct patterns of results in a series of PFTs [17]. Hence, the current observation was done to relate the predicted and observed pattern of spirometry measurement among asthmatic patients.

In our observation, the incidence of asthma is higher in females with a rate of 60% and it was similar to the study done by Prajapati et al. where a higher proportion of study participants (57.7%) was of female gender. In adults, the incidence of asthma is greater among women and it is one of the major risk factors for early adulthood asthma [18]. A wide range of clinical factors predisposes asthma risk in females during adulthood, such as higher estrogen levels, single nucleotide polymorphisms in the cyclooxygenase-2 gene, socioeconomic factors, and differential access to resources such as poor nutrition and air quality [13]. In our observation, the majority of asthmatic patients are obese, which constitutes 48.3%. Likewise, in a study done by Ramasamy et al., 50% of the asthmatic subjects were obese, with a mean BMI of 34.10 kg/m2 [19]. Obesity has negative effects on the respiratory system, which imposes mass loading of the thorax and thus decreases chest wall compliance and alterations in airway resistance obesity [19].

The GINA highlights that individuals with asthma may encounter fluctuating levels of impaired pulmonary ventilation function in distinct instances, frequently presenting as a reduction in FEV1 and FEV1/FVC. Hence, pulmonary ventilation function tests serve as a crucial diagnostic tool for asthma and aid in evaluating the extent and management of the condition. In the present investigation, there is a significant decrease in the observed value of FVC (2.05 ± 0.12 vs. 2.75 ± 0.24 L/sec; p = 0.02), FEV1 (1.78 ± 0.16 vs. 2.38 ± 0.32 L/sec; p = 0.01), and FEV1/FVC (74.46 ± 17.38 vs. 83.62 ± 15.76; p = 0.01) when compared to the predicted value among the asthmatic patients. Similar to our report, Merghani et al. also showed low FEV1 (76%), FVC (67%), and FEV1/FVC (43%) in the majority of the cases [20]. The low spirometry results in the current study might be due to various factors, such as low treatment adherence, improper inhalation technique, and irregular follow-up visits to healthcare centers. In addition, the other important factor is less spirometry assessment during the follow-up visit, precisely among the individuals who have achieved significant improvement after an acute asthma attack [21].

PEFR is an easy and reliable parameter to measure airway obstruction, particularly in patients with a risk of occupational asthma. The PEFR measurement delineates the physiological function of large airways and it decreases when there is moderate to severe airway obstruction. Further, PEFR measurement displays a significant association of the clinical course of asthma with pulmonary functions and it elicits an early sign of deteriorating lung function and is thus helpful for appropriate treatment and good patient outcomes [22]. The measurement of MVV assesses the complete function of the respiratory system, including the respiratory muscles, airway resistance, and the compliance of the lungs and chest wall [22]. Similarly, in our study, there is a significant decline in observed value for PEFR (4.76 ± 0.42 vs. 5.82 ± 0.65; p = 0.03) and MVV (78.65 ± 28.45 vs. 115.87 ± 32.15; p = 0.001) as compared to the predicted value. Similarly, a report by Merghani et al. showed that the majority of the asthmatic patients (92%) displayed low PEFR (<80% predicted) [20].

As previously stated in the study, females have a higher susceptibility to asthma risk. Therefore, we conducted a comparison of demographics and PFTs based on gender. In this study, age at diagnosis is early in females as compared to males (35.76 ± 3.76 vs. 39.65 ± 4.12; p = 0.02). Likewise, previous reports indicate that adult-onset asthma becomes the dominant phenotype in women quite early, at age 40 years, but in our study, it is still early at the age of 35 years among females [23]. In addition, BMI (p = 0.001) and the incidence of obesity (p = 0.002) were higher in females as compared to males and it was significant. Similar to our report, Forte et al. also showed increased BMI in female asthmatics as compared to their male counterparts (30.2 ± 5.8 vs. 26.9 ± 4.5 kg/m2; p = 0.002) [24]. In the present study, the incidence of severe asthma according to GINA guidelines was higher in females as compared to males (76.4% vs. 56.3%) but it was not statistically significant. Likewise, in a study done by Forte et al. there was no significant difference in the severity of asthma when compared between the female and male cases (72.2% vs. 75%; p = 0.40) [24].

The comparison of the observed significance of PFT showed a significant decrease in FVC (2.15 ± 0.32 vs. 3.18 ± 0.93 L/sec; p = 0.001), FEV1 (1.62 ± 0.18 vs. 2.04 ± 0.97 L/sec; p = 0.006), FEV1/FVC (76.54 ± 17.12 vs. 81.32 ± 19.28%; p = 0.001), and MVV (71.65 ± 15.28 vs. 89.12 ± 17.43 L/min; p = 0.01) in females when associated with males. Likewise, in Forte et al.'s study, there was a significant decline in FVC (2.3 ± 0.7 vs. 3.3 ± 1; p < 0.001) and FEV1 (1.6 ± 0.6 vs. 2.2 ± 0.9 ; p < 0.001) in females as associated to males [24]. In a cross-sectional study done by Colombo et al., the baseline FEV1 (1.8 vs. 2.3 L; p < 0.0001) showed a significant decrease in females as associated with males [25].

The current investigation possesses certain limitations. Since this was a cross-sectional study, it was not possible to create a temporal sequence among gender and the variables under investigation. Furthermore, it was a single-centered observation with a low sample size.

## Conclusions

Spirometry is a reliable technique for the evaluation of airway obstruction and lung functions in asthmatic individuals. The study showed there was a marked variation between observed and predicted values among asthmatic patients. The female gender and obesity are the main risk factors observed in the study. Female gender has a greater impact on asthma severity with the decline in observed spirometry indices as compared to males. It is crucial to have a deeper understanding of gender disparities in asthma for appropriate treatment and to enhance the quality of life.
